# Promoting Diversity, Equity, and Inclusion in the VA Scientific Workforce Through Mentored Experiences

**DOI:** 10.1089/heq.2023.0014

**Published:** 2023-05-26

**Authors:** Mark Roltsch, Cendrine Robinson, Shakeria Cohen, Carol Fowler

**Affiliations:** ^1^ Diversity, Equity and Inclusion Working Group, VA Office of Research and Development, Washington, District of Columbia, USA.; ^2^ National Institute on Deafness and Other Communication Disorders, NIH, Bethesda, MD, USA.

In summer 2020, the VA Office of Research and Development (ORD) formalized its commitment to diversity, equity, and inclusion with the formation of a DEI working group (DEIWG). A group of some 20 volunteers from within ORD expressed their commitment to diversifying the VA research workforce, promoting research on minority health and health disparities, and ensuring equity in all scientific activities within our sphere of influence, including peer review.

This commitment was fueled by demographic data on the VA research workforce showing our workforce did not adequately reflect the diversity of the Veterans we serve. Moreover, ORD leadership believed that scientists and trainees from diverse backgrounds and life experiences bring a valuable mix of perspectives and talents to help address the complex health problems faced by Veterans.

The Diversity Equity and Inclusion Working Group (DEIWG), which has now been meeting biweekly for nearly 3 years, is working to fulfill its mission of diversifying the workforce by providing training and funding opportunities to investigators who are currently underrepresented in the health sciences. Most notably, the DEIWG has been expanding the range of career levels that ORD typically supports. We recognize that individuals from certain communities may face additional barriers when it comes to achieving terminal degrees. Research shows participation of underrepresented groups in summer research programs (SRPs) improve academic outcomes and has a positive effect on students pursuing terminal degrees in STEM.

Appropriately, the DEIWG launched the ORD-sponsored SRPs. In June 2022, we welcomed 115 student participants at 21 VA sites across the country. This program is a cornerstone of ORD's efforts to strengthen and diversify the VA scientific workforce by offering research experiences to Veterans, children of Veterans, and underrepresented students.

In addition, ORD expanded its funding opportunities for trainees who already possessed a terminal degree by offering Research Supplements to Promote Diversity. This mechanism provides up to 2 years of support for an early-career scientist from an underrepresented background. Each supplement awardee works with a senior funded VA mentor who assists them in preparing for a VA Career Development Award application, which will provide up to 5 years of additional support as the trainee transitions to independence. Three cohorts of awardees have been supported through this mechanism since 2021 (32 awardees in total).

DEIWG also offered a new opportunity in 2022, Research Supplements to Promote Collaborations to Enhance Diversity in VA Research, with three awardees in the first cohort. These supplements are intended to support collaborations between VA Investigators and faculty at non-VA institutions, including, but not limited to, Minority Serving Institutions.

Furthermore, we recognize that a comprehensive DEI plan must also focus on the *retention* of investigators from underrepresented and disadvantaged backgrounds. With that in mind, we have developed an intensive proposal-writing workshop for Career Development applicants. This workshop targets young investigators who were not successful on their initial Career Development Award submissions and provides them with tools and training to increase their chances for success on subsequent submissions.

Equitable peer review is another important goal. The DEIWG has committed to analyzing the demographics of our peer reviewers and seeking creative ways to increase the diversity of our panels. Moreover, we recognize the role that unconscious biases can play in peer review and the retention of a diverse scientific workforce. For instance, confirmation bias occurs when a reviewer provides a less critical review of a principal investigator (PI) because the reviewer is making a judgment based on the PI's prior work. To this end, the DEIWG has provided educational opportunities to ORD staff so that we better understand how biases affect peer review.

Finally, ORD has expanded its commitment to research on health disparities affecting minority or other vulnerable populations. We have added a statement to all Merit Research Funding Announcement to communicate to the field that research aimed at identifying, understanding, and reducing disparities is a priority for all services. Our hope is that this initiative will encourage investigators from all disciplines to consider how their work impacts Veterans from underrepresented groups. The COVID-19 pandemic has brought health inequities under laser focus, and the DEIWG stands ready to help meet this challenge.

Future initiatives in the upcoming year include Portfolio Analysis detailing inclusivity of underrepresented Veterans in research, and recruitment of underrepresented groups.

In summary, we recognize that addressing diversity, equity, and inclusion requires a multifaceted strategy that considers the many touch points where barriers exist. The DEIWG is optimistic that ORD's commitment to these values will facilitate VA's mission of delivering outstanding care and services to our nation's Veterans, and to their families and beneficiaries.

## Abbreviations Used

DEI: diversity, equity, and inclusion
DEIWG: Diversity Equity and Inclusion Working Group
ORD: Office of Research and Development
PI: principal investigator
SRP: summer research program

